# Phenotypic plasticity of two *M. oleifera* ecotypes from different climatic zones under water stress and re-watering

**DOI:** 10.1093/conphys/coaa028

**Published:** 2020-04-13

**Authors:** Cecilia Brunetti, Antonella Gori, Barbara Baesso Moura, Francesco Loreto, Federico Sebastiani, Edgardo Giordani, Francesco Ferrini

**Affiliations:** 1 National Research Council of Italy, Department of Biology, Agriculture and Food Sciences, Institute for Sustainable Plant Protection, 50019 Sesto Fiorentino, Florence, Italy; 2 Department of Agriculture, Food, Environmental and Forestry Sciences, Section Woody Plants, University of Florence, 50019 Sesto Fiorentino, Florence, Italy; 3 National Research Council of Italy, Department of Biology, Agriculture and Food Sciences, Piazzale Aldo Moro 7, 00185 Rome, Italy; 4 Department of Biology, University Federico II, 80126 Naples, Italy

**Keywords:** Ecotypes, *Moringa oleifera*, phenotypic plasticity, secondary metabolites, water stress

## Abstract

*Moringa oleifera* is a fast-growing hygrophilic tree native to a humid sub-tropical region of India, now widely planted in many regions of the Southern Hemisphere characterized by low soil water availability. The widespread cultivation of this plant worldwide may have led to populations with different physiological and biochemical traits. In this work, the impact of water stress on the physiology and biochemistry of two *M. oleifera* populations, one from Chaco Paraguayo (PY) and one from Indian Andhra Pradesh (IA) region, was studied in a screenhouse experiment where the water stress treatment was followed by re-watering. Through transcriptome sequencing, 2201 potential genic simple sequence repeats were identified and used to confirm the genetic differentiation of the two populations. Both populations of *M. oleifera* reduced photosynthesis, water potential, relative water content and growth under drought, compared to control well-watered plants. A complete recovery of photosynthesis after re-watering was observed in both populations, but growth parameters recovered better in PY than in IA plants. During water stress, PY plants accumulated more secondary metabolites, especially β-carotene and phenylpropanoids, than IA plants, but IA plants invested more into xanthophylls and showed a higher de-epoxidation state of xanthophylls cycle that contributed to protect the photosynthetic apparatus. *M. oleifera* demonstrated a high genetic variability and phenotypic plasticity, which are key factors for adaptation to dry environments. A higher plasticity (e.g. in PY plants adapted to wet environments) will be a useful trait to endure recurrent but brief water stress episodes, whereas long-term investment of resources into secondary metabolism (e.g. in IA plants adapted to drier environments) will be a successful strategy to cope with prolonged periods of drought. This makes *M. oleifera* an important resource for agro-forestry in a climate change scenario.

## Introduction

Drought is one of the most important environmental factors limiting plant productivity worldwide (Hanjra and Qureshi, 2010). An increase in the intensity and duration of drought events has been globally observed since the 1970s, and it is expected to be exacerbated because of climate change particularly in arid and semi-arid zones (Reed *et al*., 2007; Huang *et al.* 2016), where the production of several crops is also impaired by land degradation and desertification (Geist and Lambin, 2004; Garcia-Franco *et al*., 2018). In this context, there is an increasing need to study the effects of water stress on crops and short-rotation tree plantations to select specific genotypes/ecotypes able to cope with drought conditions (Condon *et al*., 2002; Verchot *et al.,* 2007; Li *et al*., 2009; Challinor *et al*., 2014).

Phenotypic plasticity is defined as the ability of a genotype to modify its morphology, physiology and biochemistry to acclimate to multiple environmental conditions (Schlichting 1986; Colicchio and Herman, 2018). Several studies suggested that plants evolved in heterogeneous or unstable environments may exhibit a high degree of plasticity of adaptive traits, thus contributing to plant fitness and survival (Bradshaw 1965; Valladares *et al*., 2000; Callaway et al., 2003; Gianoli and Gonzalez-Teuber, 2005). This is the case of *Moringa oleifera*, a species native to sub-Himalayan mountains of northern India, where the climate is characterized by a high inter-annual and intra-season variability due to the summer monsoon activity (Singh and Mal, 2014).

*M. oleifera* is a fast-growing tree widely used as food and livestock forage because of its high nutritional value (Pandey *et al*., 2011; Saini *et al*., 2016). It is classified as a hygrophilic species, with an optimum growing temperature between 25 and 35°C (Sánchez *et al*., 2006). However, this plant can be cultivated in a variety of environmental conditions and has been introduced in many arid and semi-arid regions across Africa, South America and South East Asia (Sena *et al*., 1998; Anwar *et al*., 2007; Nouman *et al*., 2014). Previous research has provided significant gains in the understanding of the physiological and biochemical responses of *M. oleifera* to water deficit (Rivas *et al*., 2013; Förster *et al*., 2015). These researches aim at expanding cultivation of *M. oleifera* in xeric environments (Araújo *et al*., 2016; Brunetti *et al*., 2018). As *M. oleifera* is now cultivated worldwide, local adaptations may have led to the formation of different genotypes better fitted to the environment of cultivation (Muluvi *et al*., 1999; Rufai *et al*., 2013).

Plants of the same species growing under different environmental conditions have shown significant differences in the production and accumulation of primary and secondary metabolites (Hamanishi *et al*. 2015; Aranda *et al*. 2018; Cotrozzi *et al*. 2018). The study of these intra-specific variations is very useful in the characterization of populations, which are collected from different regions. In particular, the identification of physiological and biochemical traits that are specifically affected by water stress and their plasticity may help further improve understanding of mechanisms of *M. oleifera* acclimation. The high plasticity of stomatal conductance and secondary metabolism may allow this species to maximize growth and physiological performances during the favourable season and, at the same time, to overcome drought periods by up-regulating the biosynthesis of multiple secondary metabolites, such as isoprenoids and flavonoids, thus limiting severe photo-inhibitory processes and oxidative damages (Brunetti *et al*., 2018). In addition, selection of populations with high phenotypic plasticity may sustain agricultural breeding programs in arid and semi-arid environments (Yordanov *et al.*, 2000).

In this study, we compared the responses to water stress and re-watering of two populations of *M. oleifera* from a humid sub-tropical and a tropical wet-dry zone. These two populations do not present distinct morphological differences, however, considering the multiple biochemical and physiological mechanisms that allow *M. oleifera* to tolerate drought episodes (Brunetti *et al*., 2018) and the extensive genetic variability reported in Indian *M. oleifera* populations (Kumar Ganesan *et al*., 2014; Rajalakshmi *et al*., 2018), we hypothesized a biochemical divergence in these two *M. oleifera* populations when exposed to water stress.

Specifically, our work aimed to (i) identify and quantify genetic, physiologic and metabolic differences in *M. oleifera* plants originated from two different natural populations grown under water stress conditions and re-watering and (ii) use traits of the two populations as terms of reference to select *M. oleifera* plants adapted to drought conditions.

## Material and methods

### Plant material and water stress treatment

Seeds were obtained from two populations of *M. oleifera* Lam. of different origins. The first population (PY) was from the Chaco Paraguayo region, an area situated north-west of the city of Asunción, Paraguay (25°18′S 57°38′W). Asunción has a humid subtropical climate (Köppen: Cfa) characterized by hot, humid summers (average of 27.5°C in January) and mild winters (average of 17.6°C in July). The mean annual temperature is 22.7°C, while the mean annual precipitation is 1420 mm, with most rainfall in April (https://en.climate-data.org/). The second population (IA) was from an area close to Visakhapatnam, in the region of Andhra Pradesh, India (17°42′N 83°17′E). The climate of this region is tropical wet and dry (Köppen: Aw). The mean annual temperatures are around 27°C, with the maximum in the month of May (31.2°C) and the minimum in January (23.5°C). In a year, the mean annual precipitation is 1008 mm, concentrated in the monsoon season. The driest month is December, with 6 mm of rainfall, while precipitations peak in October, with an average of 260 mm (https://en.climate-data.org/). In both sites, seeds were collected from local cultivations in small farms.

The study was conducted at the University of Florence in an experimental plot located in Sesto Fiorentino (Florence, Italy) (43°49′N 11°12′E). Seeds from the two provenances were sown and then transplanted in June 2012. Six-week-old seedlings were potted into 6 l containers and grown outdoor until the beginning of the experiment. Substrate was a mixture of peat and lapillus (3:1, v:v) with addition of a fertilizer (Osmocote Pro 3–4 m, Everris, The Netherlands). Two weeks before the beginning of the experiment, 30 plants per population were moved to screenhouses (6 m × 4 m × 2 m, length × width × height) without walls and with a roof of ETFE fluoropolymer film (NOWFLON, ET-6235, Kunststoffprodukte GmbH & Co. KG, Siegsdorf, Germany) to acclimate [26 ± 2°C/19 ± 1°C day/night temperature; 32.7 ± 2°C mean midday temperature; 800 ± 100 μmol m^−2^ s^−1^ photosynthetic photon flux density (PPFD) at midday]. In each screenhouse, both populations were divided in four groups of 6 plants named WS (water-stressed plants), R-WS (re-watered plants), WW (well-watered plants used as control for WS plants) and R-WW (well-watered plants used as control for re-watered plants, that is, grown for a period similar to R-WS and longer than WW and WS). During the acclimation period, plants were hand watered regularly to pot capacity, then water stress was imposed by withholding water from WS and R-WS plants until the stomatal conductance (*g*_s_) reached 5% of *g*_s_ measured in WW plants (10 days after water withholding started).

At the water stress endpoint, physiological characterization of WS and WW plants was carried out, and WS and WW plants were harvested for biochemical analyses while R-WS plants were re-watered to pot capacity. Physiological and biochemical measurements were repeated after a 10-day recovery on both R-WS and R-WW plants, after assessing the full recovery of photosynthesis and stomatal conductance in one of the two populations. Temperature was recorded using a data logger system (Tinytag Ultra2, Gemini Dataloggers UK). All physiological and biochemical measurements were performed on four replicates per population and per group.

### Transcriptome analysis and molecular markers development

Highly pure total RNA was extracted from leaves of the two populations of *M. oleifera* using the method described in Chang et al. (1993). Two RNA-Seq libraries, corresponding to the two populations, were prepared using Illumina TruSeq RNA sample Prep Kit (Illumina, Inc., CA, USA). cDNA libraries construction and paired-end (2 × 100) sequencing, using Illumina HiSeq2000 system (Illumina, Inc.) was performed at IGA Technology Services S.r.l. (Udine, Italy). All sequence data were deposited at the National Center for Biotechnology Information Sequence Read Archive under BioProject PRJNA (510938). The reads were processed as detailed in Torre et al. (2016) and combined to produce a *de novo* assembly using Trinity v. 20 130 225 (Haas et al., 2013) with default k-mer size of 25. Transcripts longer than 200 bp were selected and clustered at 95% identity using CD-HIT-EST v4.6.1 (Fu et al., 2012). Transcripts were annotated through BlastX sequence comparison to the plant UniProt database with an E-value threshold of 1e^−10^. In addition, two transcriptomes were assembled separately for each population by using CLC genomics workbench as detailed in Torre et al. (2014). MISA software (http://pgrc.ipk-gatersleben.de/misa/) was used to identify simple sequence repeats (SSRs) in each of the *de novo* assembled transcriptomes of *M. oleifera*.

### Gas exchanges and chlorophyll fluorescence

Gas exchanges were measured using a LI-6400 portable photosynthesis system (Li-Cor, Lincoln, NE, USA). Measurements were performed at a PPFD of 1000 μmol photons m^−2^ s^−1^, 400 ppm of CO_2_ and ambient temperature (31 ± 1°C). Photosynthesis (*P*_n_) and g_s_ were calculated using the LI-6400 software. Responses of P_n_ to C_i_ were generated by changing CO_2_ concentration between 50 and 1400 μmol mol^−1^ after removing stomatal limitation (Centritto *et al*., 2003). The method of Sharkey *et al*. (2007) was used to fit the curves and calculate the apparent maximum carboxylation rate allowed by Rubisco (Vc_max_). Chlorophyll fluorescence was measured using an imaging PAM chlorophyll fluorometer (Heinz Walz, Effeltrich, Germany). Minimum fluorescence (F_0_) was measured with a 0.8 μmol m^−2^ s^−1^ light beam on dark-adapted leaves. Maximum fluorescence in the dark-adapted state (F_m_) was determined using saturating pulses (0.5 s) of red light (8000 μmol m^−2^ s^−1^), thus allowing calculation of F_v_/F_m_ (F_v_/F_m_ = (F_m_—F_0_)/F_m_). Actinic red continuous light (1000 μmol m^−2^ s^−1^) was then switched on, and steady-state fluorescence was recorded (F_s_). Saturating pulses were then applied to record the maximum fluorescence under actinic light (F’_m_). These data were used to calculate the actual quantum yield of PSII (Φ_PSII_ = (F’_m_—F_s_)/F’_m_, Genty *et al*., 1989). Electron transport rate (ETR) was calculated with the following equation:

$$\begin{equation*} \textrm{ETR} = 0.5\times \Phi_\textrm{PSII} \times \textrm{PAR} \times 0.85\end{equation*}$$

where 0.5 was a factor assuming an equal distribution of photons between PSI and PSII and 0.85 was the leaf absorbance estimated with a Li-Cor 1800 spectroradiometer equipped with a Li-Cor 1800-125 integrating sphere.

### Water status and growth

Water potential (*Ψ*w, MPa) was measured using a Scholander-type pressure-chamber (PMS Instruments, Corvallis, OR) according to Scholander *et al*. (1965). Osmotic potential (*Ψ*π, MPa) was measured on the sap of frozen and thawed leaves, using a boiling point VAPRO 5520 osmometer (Wescor Inc., South Logan, UT) (Ball and Oosterhuis 2005). Water potential and osmotic potential were measured at predawn, between 4:00 am and 6:00 am on two leaves per plant. Leaf turgor loss point (π_tlp_) was calculated from osmotic potential applying the formula reported in Bartlett *et al.* (2012a):

$$\begin{equation*} \pi_{\textrm{tlp}} = 0.832^{\ast}\Psi\pi- 0.631\end{equation*}$$

The relative water content (RWC, %) was measured on fully developed leaves using the following equation:

$$\begin{equation*} \textrm{RWC} (\% ) = (\textrm{FW} - \textrm{DW})^{\ast}100/(\textrm{TW}-\textrm{DW})\end{equation*}$$

where FW, DW and TW are the fresh, the dry and the turgid mass of the leaf, respectively. Fresh mass was measured at predawn, immediately after excising the leaf from the plant. After FW determination, leaves were placed in vials filled with de-ionized water, sealed with a plastic bag and placed in the dark for 24 h. After rehydration, leaves were weighed and TW determined. Then, leaves were oven-dried for 72 h at 80°C and weighed again to determine the DW.

Leaf water and osmotic potential and RWC were measured in the same days of the other physiological measurements and collection of samples for biochemical analyses.

Shoot relative growth rate (RGR, cm day^−1^) and leaf area expansion rate (LAER, cm^2^ day^−1^) were calculated by measuring the increments in shoot length (Gratani *et al.*, 2003) and the newly developed leaf area (calculated with the software ImageJ) on a diurnal basis (Guidi et al., 2008), respectively.

### Quantification of non-structural carbohydrates

Soluble carbohydrates as sucrose, glucose, fructose and galactose were identified and quantified by a high-performance liquid chromatography (HPLC) analysis. In detail, 75 mg of lyophilized tissue were extracted with 3 × 5 ml of 75% ethanol, the ethanol fraction reduced to dryness under vacuum, and finally rinsed with 2 ml of water (pH 7). The extract was purified using -CH and -SAX Bond-Elute cartridges (Agilent, Santa Clara, CA, USA) and the eluate reduced to dryness. Samples were rinsed with ultrapure water, injected in a Series 200 HPLC equipped with 200-RI detector (all from Perkin-Elmer, Bradford, CT, USA) and separated on an 8 × 300 mm SC1011 column (Showa Denko, Tokyo, Japan) maintained at 88 ± 1°C. Eluent was ultrapure water at a flow rate of 0.8 ml min^−1^.

### Quantification of phenylpropanoids

Lyophilized leaf material (100 mg) was extracted three times with a mixture of 75% EtOH/25% H_2_O acidified to pH 2.5 with HCOOH. The supernatant was partitioned with 3 × 5 ml of *n*-hexane, reduced to dryness and rinsed with 1.5 ml of CH_3_OH/H_2_O (8:2). Aliquots of 5 μl were injected into a Perkin Elmer Flexar liquid chromatograph equipped with a quaternary 200Q/410 pump and an LC 200 diode array detector (all from Perkin Elmer, Bradford, CT, USA). Phenylpropanoids were separated using a 150 × 4.6 mm Waters (Waters Italia, Milan, Italy) Sun Fire column (5 *μ*m) operating at a flow rate of 1 ml min and a temperature of 25°C and. The mobile phases were (A) H_2_O [added with CH_3_COONH_4_ (10 mM) pH 4.3 with CH_3_COOH]/CH_3_CN (95/5, v/v) and (B) H_2_O [added with CH_3_COONH_4_ (10 mM) pH 4.3 with CH_3_COOH]/CH_3_CN (5/95). Phenylpropanoids were separated using a gradient elution from A to B over a 46-min run and identified on the basis of their retention times, UV spectral characteristics of authentic standards (Extrasynthese, Lyon-Nord, Genay, France). The phenylpropanoid pool consisted of caffeic acid, apigenin, quercetin and kaempferol derivatives, all reported as μmol g^−1^DW.

### Quantification of photosynthetic pigments

Individual carotenoids were identified and quantified in four leaves collected from four different plants at pre-dawn (6:00 am) and mid-day (12:00). The lyophilized leaf material (120 mg) was extracted with 2 × 5 ml acetone (added with 0.5 g l^−1^ CaCO_3_) and injected (15 μl) into the Perkin Elmer liquid chromatography unit reported in the previous paragraph. Photosynthetic pigments were separated using a 250 × 4.6 mm Agilent Zorbax SB-C18 (5 μm) column operating at 30°C and at a flow rate of 0.8 ml min^−1^. The elution was obtained with 18-min linear gradient solvent passing from 100% CH_3_CN/MeOH (95/5 added with 0.05% of triethylamine) to 100% MeOH/ethyl acetate (6.8/3.2). Lutein (μmol/g DW), β-carotene (μmol/g DW), zeaxanthin (μmol/g DW), anteraxanthin (μmol/g DW), violaxanthin (μmol/g DW), neoxanthin (μmol/g DW), xanthophyll cycle pigments [violaxanthin (V), anteraxanthin (A), zeaxanthin (Z), collectively named VAZ], chlorophyll a and chlorophyll b were identified and quantified using visible spectral characteristics and retention times of authentic standards (all from Extrasynthese, Lyon-Nord, Genay, France). The de-epoxidation state of xanthophylls (DES) was calculated according to the formula:

$$\begin{equation*} \textrm{DES} = (0.5\ \textrm{A}+\textrm{Z})/(\textrm{V}+\textrm{A}+\textrm{Z}).\end{equation*}$$

### Quantification of ABA and ABA-GE

The quantification of abscisic acid (ABA) and ABA-glucose ester (ABA-GE) was performed utilizing lyophilized leaf tissue (150 mg) added with 40 ng of d_6_-ABA and 40 ng of d_5_-ABA-GE. The extraction was performed with 3.5 ml of CHCl_3_/CH_3_OH/H_2_O (12:5:1) at 4°C for 30 minutes. The supernatant was partitioned with 2.9 ml of CHCl_3_ and 1.35 ml of H_2_O (pH 8). The aqueous-methanolic fraction was collected, acidified at pH 2.5 with HCOOH, loaded onto the Sep-Pak C18 cartridges and washed with 2 ml acidified water (pH 2.5). ABA and ABA-GE were eluted with 2 ml of acetone/CH_3_OH (1/1). The eluate was dried under nitrogen and rinsed with 500 μl CH_3_OH/H_2_O 50/50 pH 2.5. Identification and quantification of ABA and ABA-GE was performed injecting 3 μl in a Agilent LC1200 chromatograph coupled with an Agilent 6410 triple quadrupole MS detector equipped with an ESI source (all from Agilent Technologies, Santa Clara, CA). Analyses were done in negative ion mode. Compounds were separated in a Poroshell C18 column (3.0 × 100 mm, 2.7 μm i.d., Agilent) using a binary solvent system with water (added with 0.1% of HCOOH, solvent A) and acetonitrile/methanol (1/1) (added with 0.1% of HCOOH, solvent B). The solvent gradient was programmed to change linearly from 95% A to 100% B during a 30-min run at a flowrate of 0.3 ml min^−1^. Quantification was conducted in multiple reaction mode (López-Carbonell *et al*., 2009).

### Statistics and data analysis

Data were analysed using a repeated-measures analysis of variance (ANOVA) (Girden, 1992), where `ecotype’ and `water treatment’ were fixed between-subjects effects and `sampling time’ (at water stress end-point and at re-watering) was the within-subjects effect. For parameters showing significant interactions, differences between treatments were analyzed using one-way ANOVA (SPSS v.20; IBM, Chicago IL, USA). Significant differences among means (*n* = 4) were estimated at the 5% (*P* < 0.05) level, using Tukey’s test.

The extent to which the examined traits (X) underwent adjustments because of water stress (NIV_WS_) or re-watering (NIV_R-WS_) was determined based on a normalized index of variation (NIV; Guidi *et al*., 2008), calculated as follows:

$$\begin{align*} \textrm{NIV}_{\textrm{WS}} =&\ (\textrm{X}_{\textrm{WS}} - \textrm{X}_{\textrm{WW}})/ (\textrm{X}_{\textrm{WS}} + \textrm{X}_{\textrm{WW}})\\ \textrm{NIVR}_{\textrm{-WS}} =&\ (\textrm{X}_{\textrm{R-WS}} - \textrm{X}_{\textrm{R-WW}})/ (\textrm{X}_{\textrm{R-WS}} + \textrm{X}_{\textrm{R-WW}}).\end{align*}$$

The NIV was used to determine the degree of phenotypic plasticity for each trait in the two genotypes of *M. oleifera*, in detail high NIV values indicate higher plasticity and lower NIV values indicate lower plasticity.

## Results

### Genetic characterization of the two populations of *M. oleifera*

A preliminary genetic analysis was conducted on the two provenances through transcriptome comparison. A total of 79 499 242 sequencing reads from PY and IA populations were obtained after quality filtering. The sequences from both populations were clustered to a final data set of 63 174 transcripts (10.6084/m9.figshare.11898141), out of which 50 879 (80.5%) were annotated. Microsatellites loci identified in the two populations were *in silico* compared: out of 2201 SSRs mapped in the two populations, 186 were polymorphic, although presence of heterozygosities could not be ruled out. Finally, a total of 11 509 variants were identified including deletions, insertions, inversions and translocations, thus indicating that plants of PY populations and plants of IA populations can be genetically distinguished and can be identified as two different ecotypes.

### Physiological responses induced by water stress and re-watering

**Figure 1:**
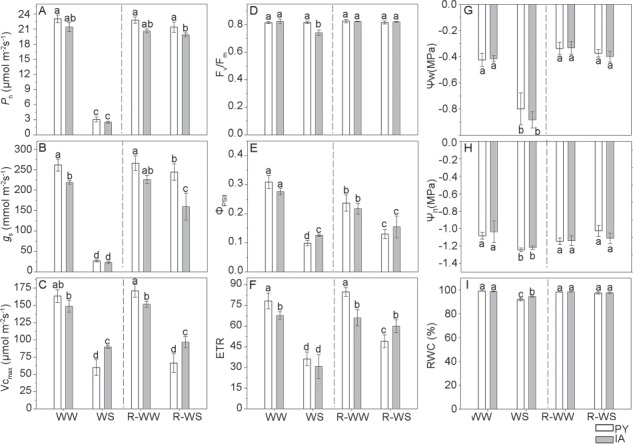
Net photosynthesis (*P*_n_), stomatal conductance (*g*_s_), apparent maximum rate of carboxylation (Vc_max_), maximal efficiency of PSII photochemistry (F_v_/F_m_), actual efficiency of PSII (Φ_PSII_), ETR, water potential (*Ψ*_w_, MPa), osmotic potential (*Ψ*_π_, MPa) and RWC in two *M. oleifera* ecotypes (PY ecotype from Paraguay and IA ecotype from India) comparing well-watered plants (WW) with plants at the end of water stress (WS) and well-watered plants (R-WW) with plants subjected to re-watering (R-WS). Data are means ± standard deviation (*n* = 4). Different letters represent significant differences between ecotypes and treatments (*P* < 0.05).

Under well-watered conditions (WW and R-WW), the two ecotypes showed different photosynthetic traits, with higher values of *g*_s_ and Vc_max_ compared to IA plants (Fig. 1). *P*_n_ and *g*_s_ were reduced by water stress in both ecotypes (Fig. 1A and B). After re-watering, *P*_n_ completely recovered in all plants, while g_s_ remained lower in IA compared to PY plants (Fig. 1A and B). Vc_max_ was lower in PY than in IA plants both under water stress and re-watering conditions (Fig. 1C).

The maximum efficiency of photosystem II (F_v_/F_m_) did not change during the whole experiment in PY plants, while in IA plants F_v_/F_m_ significantly decreased under stress, and fully recovered after re-watering (Fig. 1D). In WS plants of both ecotypes, the actual efficiency of photosystem II (Φ_PSII_) declined of ~ 60%, while the ETR declined of ~ 50%. These two parameters recovered slightly better in IA than in PY plants after re-watering, although the recovery was incomplete in both plants (Fig. 1E and F).

Under water stress, the two ecotypes showed a similar reduction in water potential (*Ψ*_w_), osmotic potential (*Ψ*_π_) and leaf turgor loss point (π_tlp_) compared to well-watered plants (Fig. 1G and H; Supplementary Table S1); RWC decreased more in PY plants than in IA plants (Fig. 1I). After re-watering, all indicators (*Ψ*_w_, *Ψ*_π_, π_tlp_ and RWC) fully recovered in both ecotypes (Fig. 1G–I; Supplementary Table S1).

**Figure 2:**
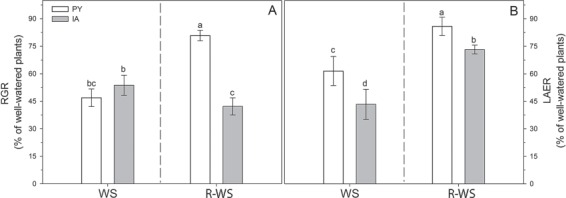
Shoot RGR and LAER in two *M. oleifera* ecotypes (PY ecotype from Paraguay and IA ecotype from India) at the end of water stress (WS) and subsequent re-watering (R-WS) period reported as percentage of the corresponding well-watered plants (WW and R-WW, respectively). Data are means ± standard deviation (*n* = 4). Different letters represent significant differences between ecotypes and treatments (*P* < 0.05).

Water stress inhibited shoot RGR and LAER in both ecotypes (Fig. 2). However, LAER was higher in water-stressed PY plants compared to water-stressed IA plants (Fig. 2B). After re-watering, RGR increased only in PY plants (Fig. 2A), while LAER recovered in both ecotypes and almost reached the values of well-watered plants in PY ecotype (Fig. 2B).

### Biochemical responses induced by water stress and re-watering

**Figure 3:**
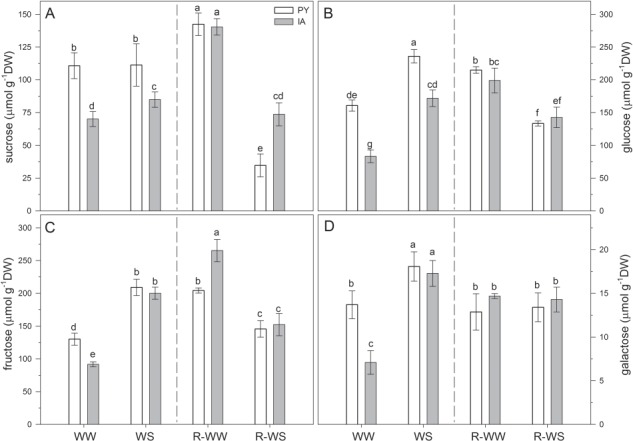
Content of soluble carbohydrates (sucrose, glucose, fructose and galactose) in two *M. oleifera* ecotypes (PY ecotype from Paraguay and IA ecotype from India) comparing well-watered plants (WW) with plants at the end of water stress (WS) and well-watered plants (R-WW) with plants subjected to re-watering (R-WS). Data are means ± standard deviation (*n* = 4). Different letters represent significant differences between ecotypes and treatments (*P* < 0.05).

Soluble carbohydrates are synthesized in response to drought and help plants to cope with osmotic stress. In WW leaves soluble carbohydrates (sucrose, glucose, fructose and galactose) were higher in PY leaves than in IA leaves (Fig. 3A–D). However, at the end of experimental period (20 days), differences in soluble carbohydrates content between well-watered plants (R-WW) of the two ecotypes were observed only for fructose (higher in IA than in PY, Fig. 3C). Water stress increased the accumulation of glucose, fructose and galactose in both ecotypes (Fig. 3B–D). Sucrose increased significantly only in IA plants under water stress (Fig. 3A). There were no significant differences in monosaccharides content (glucose, fructose and galactose) between the two ecotypes after re-watering (Fig. 3B–D), whereas sucrose of PY R-WS plants was lower than in all other treatments (WW, WS and R-WW) and also lower than in IA plants (Fig. 5A). All soluble carbohydrates, excepted galactose, were lower in R-WS plants of both ecotypes compared to the corresponding R-WW counterparts.

**Figure 4:**
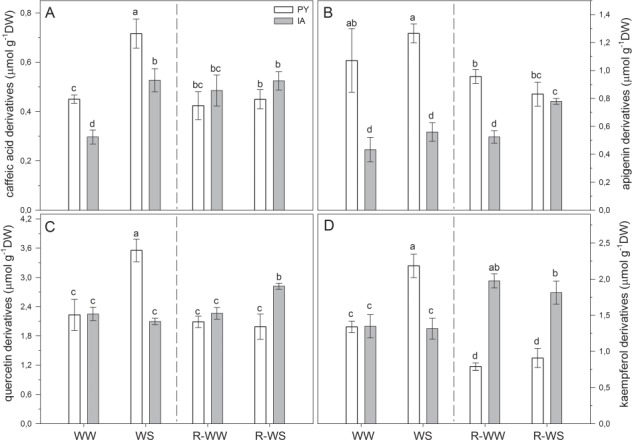
Content of phenylpropanoids (caffeic acid derivatives, apigenin derivatives, quercetin derivatives and kaempferol derivatives) in two *M. oleifera* ecotypes (PY ecotype from Paraguay and IA ecotype from India) comparing well-watered plants (WW) with plants at the end of water stress (WS) and well-watered plants (R-WW) with plants subjected to re-watering (R-WS). Data are means ± standard deviation (*n* = 4). Different letters represent significant differences between ecotypes and treatments (*P* < 0.05).

Phenylpropanoids are synthetized in response to multiple abiotic stresses, including drought, and have a prominent role to counter photo-oxidative damage in leaves. In well-watered plants (WW and R-WW), the content of the different phenylpropanoids changed during the experimental period (Fig. 4). Caffeic acid and apigenin derivatives were higher in PY than in IA plants in WW and WS conditions; however, this difference was reproduced only for apigenin derivatives in R-WW leaves (Fig. 4A and B). The most abundant phenylpropanoids present in *M. oleifera* leaves were quercetin derivatives, which did not differ between PY and IA plants under well-watered conditions (WW and R-WW plants, Fig. 4C). PY plants showed higher content of kaempferol derivatives than IA plants only in WS, while a higher content of these compounds was found in IA plants under both R-WW and R-WS conditions (Fig. 4D).

When plants were exposed to water stress, caffeic acid derivatives increased in both ecotypes (+59% and +77% in PY and IA plants, respectively; Fig. 4A), while a significant increment in kaempferol (+59%) and quercetin (+63%) derivatives was observed only in PY plants (Fig. 4C and D). After re-watering, an increment in apigenin (+ 49%) and quercetin (+25%) derivatives was observed in IA compared to well-watered plants (Fig. 4B–C). By contrast, in PY re-watered plants all phenylpropanoids declined with respect to WS plants, reaching values similar to or lower than those of WW plants (Fig. 4A–D).

**Figure 5:**
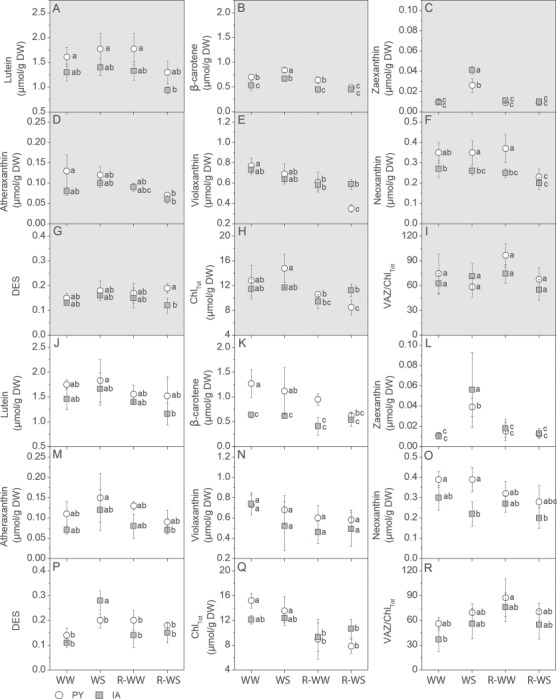
Photosynthetic pigments measured at (A–I) pre-dawn and (J–R) mid-day: lutein (A, J), β-carotene (B, K), zeaxanthin (C, L), anteraxanthin (D,M), violaxanthin (E, N), neoxanthin (F, O), xanthophyll cycle pigments (violaxanthin (V), anteraxanthin (A), zeaxanthin (Z), collectively named VAZ, G, P), de-epoxidation state of xanthophylls (DES, H, Q), total chlorophyll (Chl_Tot_, I, R) and the ratio VAZ on total chlorophyll (VAZ/Chl_Tot_, I,R) in two *M. oleifera* ecotypes (PY ecotype from Paraguay and IA ecotype from India) comparing well-watered plants (WW) with plants at the end of water stress (WS) and well-watered plants (R-WW) with plants subjected to re-watering (R-WS). Data are means ± standard deviation (*n* = 4). Different letters represent significant differences between genotypes and treatments (*P* < 0.05).

The biochemical compounds synthetized in the MEP (methylerythritol phosphate pathway) include both carotenoids and hormones, such as ABA. These metabolites play essential roles in response to external environmental stimuli, protecting PSII photochemistry and preventing water loss by stomata closure. The two ecotypes did not differ on photosynthetic pigments under well-watered conditions (Fig. 5), except for β-carotene content, which was higher in PY ecotype compared to IA ecotype, especially at midday (Fig. 5). Water stress increased zeaxanthin levels both under pre-dawn and midday conditions while midday de-epoxidation state of xanthophylls (DES) was also slightly stimulated in both ecotypes (Fig. 5). These effects were significantly higher in IA than in PY plants. In addition, under water stress, the content of neoxanthin was lower in IA plants than in PY plants at midday. Lutein, antheraxanthin and total chlorophylls (Chl_tot_) were not significantly affected by water stress and re-watering in both ecotypes. Total chlorophylls (Chl_tot_) were always higher in IA than in PY plants after re-watering.

**Figure 6:**
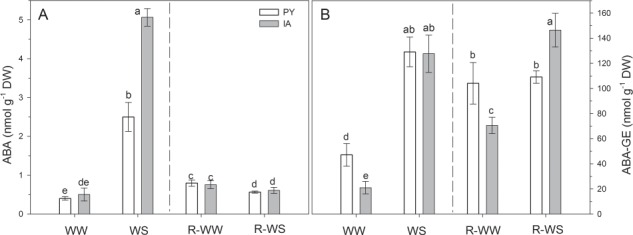
Content of ABA and ABA-GE in two *M. oleifera* ecotypes (PY ecotype from Paraguay and IA ecotype from India) comparing well-watered plants (WW) with plants at the end of water stress (WS) and well-watered plants (R-WW) with plants subjected to re-watering (R-WS). Data are means ± standard deviation (*n* = 4). Different letters represent significant differences between ecotypes and treatments (*P* < 0.05).

Under well-watered conditions (WW and R-WS), no differences were observed in ABA content between the two ecotypes (Fig. 6A), while ABA-GE was higher in PY than in IA plants (Fig. 6B). Water stress boosted ABA biosynthesis in both ecotypes; however, the increment in ABA content was higher in IA than in PY plants (Fig. 6A). After re-watering, ABA decreased in both ecotypes, reaching values slightly lower than in WW plants (Fig. 6A). ABA-GE increased in WS plants of both ecotypes and after re-watering returned to values similar to WW only in PY plants (Fig. 6B).

### Plasticity

The NIV showed different levels of plasticity in the two ecotypes of *M. oleifera* for the examined physiological and biochemical traits (Table 1). Among physiological parameters, the main differences in NIV values between ecotypes were observed for Vc_max_ and Φ_PSII_. In detail, NIV values of Vc_max_ and Φ_PSII_ varied much more in PY than in IA under water stress, as well as upon re-watering (Table 1). The phenylpropanoid content (phenylpropanoids_tot_), resulting from the sum of all phenylpropanoids quantified in the leaves of *M. oleifera*, increased in response to water stress mainly in PY (NIV = 0.20). By contrast, the xanthophylls pool (mean NIV = 0.39) and DES (NIV = 0.50) were greatly affected by water stress in IA, mainly because of variations in zeaxanthin (NIV = 0.71) (Table 1).

**Table 1:** NIV of the physiological and biochemical traits for two *M. oleifera* ecotypes (PY ecotype from Paraguay and IA ecotype from India) for water stress (NIV_WS_) and re-watering (NIV_R-WS_) conditions

Trait	NIV_WS_ PY	NIV_WS_ IA	NIV_R-WS_ PY	NIV_R-WS_ IA
Ψ_w_	0,31	0,36	0,05	0,09
Ψ_π_	0,07	0,08	−0,05	−0,05
RWC	−0,04	−0,02	0,00	0,00
*P*_n_	−0,77	−0,79	−0,03	−0,02
g_s_	−0,81	−0,81	−0,04	−0,17
Vc_max_	−0,46	−0,25	−0,44	−0,22
F_v_/F_m_	0,00	−0,05	−0,01	0,00
ϕ_PSII_	−0,52	−0,37	−0,29	−0,17
ETR	−0,37	−0,38	−0,27	−0,05
ABA	0,83	0,82	−0,19	−0,42
ABA-GE	0,47	0,72	0,02	0,35
Carbohydrates tot	0,12	0,09	−0,27	−0,22
Phenylpropanoids tot	0,20	0,02	−0,02	0,06
Violaxanthin (V)	−0,09	−0,26	−0,06	−0,15
Antheraxanthin (A)	0,03	0,21	−0,10	−0,02
Zeaxanthin (Z)	0,55	0,71	−0,08	−0,23
β-Carotene	−0,17	−0,02	0,09	0,17
DES	0,17	0,50	−0,03	0,06
RGRs	−0,40	−0,30	−0,11	−0,41
LAER	−0,24	−0,39	−0,08	−0,16

## Discussion

### Genetic differences of *M. oleifera* plants from different climatic zones

In the present study, we compared the responses to water stress and re-watering of two *M. oleifera* populations: one from the humid sub-tropical region of Chaco in Paraguay and the other from the wet and dry climate region of Andhra Pradesh in India. Our genetic analysis revealed that the two populations developed significant genetic differences. In particular, the two populations showed a high level of genetic differentiation as estimated *in silico* by the variation at multiple SSR loci and had the same magnitude of those observed in two plant guar varieties (Tanwar et al., 2017). Although adaptation to local condition cannot be excluded, since the spread of the species is quite recent, the genetic diversity highlighted in the two provenances is more likely due to the high genetic diversity of the species in the site of origin. In fact, preliminary studies (Rufai *et al*., 2013; Kumar Ganesan *et al.* 2014; Rajalakshmi *et al*. 2018) indicate a high level of genetic variation of *M. oleifera* in north-western India, in its natural distribution area, even though a deep and detailed knowledge of the distribution of genetic diversity of this species is still lacking. On the basis of this genetic differences and the scientific literature (Hufford and Mazer 2003; Bakhtiari *et al*. 2019), we call the two populations ecotypes and surmise that biochemical and physiological differences in the response to water stress may be genetically fixed in the two ecotypes.

### Ecotypic differences are exacerbated by water-stress and re-watering. I: physiology and growth

Under well-watered conditions, the two ecotypes of *M. oleifera* showed only slight physiological differences. In WW plants, these included a higher stomatal conductance in PY compared to IA. However, in R-WW, also *P*_n_, Vc_max_ and ETR were significantly lower in IA than in PY plants. Also ABA-GE and β-carotene content were lower in IA than in PY plants at the R-WW stage, taken together this may represent an early onset of senescence in IA than in PY plants. Photosynthates (soluble sugars) and some secondary metabolites (caffeic acid and apigenin derivatives) were higher in PY plants than in IA plants, especially at the WW stage, confirming higher photosynthetic capacity of PY plants.

Significant physiological differences between the two ecotypes clearly emerged after the water stress treatment, as observed also in other tree species (Rieger *et al*., 2003; Deluc *et al*., 2009; Hamanishi *et al*., 2015). Water stress decreased *P*_n_ and *g*_s,_ to a similar extent in both ecotypes (Fig. 1A and B); however, both ecotypes of *M. oleifera* maintained relatively high values of RWC under water stress, confirming that this is a isohydric dehydration-sensitive species (Brunetti *et al*., 2018). Indeed, water stress led to a similar reduction of π_tlp_ in both ecotypes, which did not show *high* `safety margins’ for avoiding turgor loss; however, a quick loose of turgor may have allowed a prompt stomatal closure thereby maintaining a high RWC (Bartlett *et al.*, 2012b; Henry *et al.,* 2019). As consequence, a reduction in *g*_s_ induced a strong downregulation of photosynthesis due to stomatal closure and reduced CO_2_ availability (Medrano *et al.*, 2002). In our experiment, water stress was particularly severe and stomatal limitations were accompanied by biochemical limitations, as shown by the significant reduction in Vc_max_ in both ecotypes, further reducing photosynthesis (Fig. 1C). Vc_max_ decline was particularly strong in PY plants, indicating that ribulose 1,5- bisphosphate (RuBP) carboxylase-oxygenase (Rubisco) activity or in RuBP regeneration could be more affected in this ecotype (Makino *et al.*, 1985; Centritto *et al*., 2003). Water stress also impaired the actual efficiency of PSII (Φ_PSII_) and ETR in both ecotypes (Fig. 1D and E). This is consistent with the observed inhibition of photosynthesis and of increasing biochemical and diffusional limitations under water stress (Flexas *et al*., 2004). However, we also observed a 25% reduction of F_v_/F_m_ in water-stressed IA plants, indicating that the photochemistry of this ecotype is more susceptible than PY photochemistry to water stress. The reduction in F_v_/F_m_ is attributable to an increase in initial chlorophyll fluorescence (F_0_, *data not shown*), indicating a physical dissociation of light harvesting complex from PSII and a block of energy transfer to PSII traps (Armond *et al*., 1980; Havaux, 1993). Interestingly, photosynthetic limitations, despite being different in the two ecotypes, eventually resulted in similar reduction of RGR due to water stress (Fig. 2). However, the LAER was more curbed in water-stressed IA plants, suggesting that photochemical limitations had a stronger impact on the phenotype of water-stressed *M. oleifera*. This observation awaits for more compelling experimental confirmation.

Physiological divergences between the two ecotypes were also observed upon re-watering. In particular, the recovery of plant growth (RGR, LAER) (Fig. 2), photosynthesis and stomatal conductance (Fig. 1A, B) was more complete in PY than in IA plants. Interestingly, however, the complete recovery of photosynthesis after re-watering was not accompanied by a similar recovery of biochemical and photochemical efficiencies, as Vc_max_ and Φ_PSII_ remained lower in re-watered plants of both ecotypes compared to their well-watered counterparts (Fig. 1C and E). This indicates that recovery of photosynthesis under ambient conditions might be due to attenuation of CO_2_ diffusive limitations that limited photosynthesis under water stress (Flexas *et al*., 2006).

### Ecotypic differences are exacerbated by water-stress and re-watering. II: biochemistry and metabolism

The two ecotypes of *M. oleifera* showed a series of interesting metabolic differences already under well-watered conditions. PY produced more soluble sugars than IA plants, despite photosynthesis being similar in WW plants of the two ecotypes (Fig. 3). We did not measure starch, but our observations may indicate a different partitioning between soluble and non-soluble sugars of photosynthates produced by the two ecotypes (Sharkey, 2015). These differences were not retrieved in R-WW plants that were sampled 10 days later, and so we speculate that this effect may be developmentally controlled, being only observed in leaves that must sustain rapid growth. PY plants also produced more β-carotene and higher levels of some (albeit not the most common) phenylpropanoids compared to IA plants in WW and R-WW conditions. This indicates that in PY plants more carbon is diverted into secondary metabolites, potentially helping plant resilience after stress because of their antioxidant activity against reactive oxygen species (Tattini *et al.,* 2015). However, β-carotene content of PY and IA leaves dropped at levels similar at the R-WW stage and does not seem to have contributed to better photoprotection in PY leaves, as discussed earlier.

The two ecotypes of *M. oleifera* also showed different biochemical adjustments when exposed to water stress. Water stress stimulated an increase in monosaccharides in both ecotypes compared to well-watered conditions (Fig. 3). Accumulation of readily metabolized carbohydrates (e.g. glucose, fructose and galactose) is common in leaves of plants exposed to short-term water deficit, as soluble sugars are excellent osmolytes (Bruce *et al*., 2007). In our experiment, the osmotic potential was indeed affected by water stress and soluble sugars might have acted as osmoprotectants of membranes and proteins in cells exposed to water stress, reducing aggregation of denatured proteins (Ashraf and Harris, 2003). Perhaps the higher content of soluble sugars in PY plants under water stress could have helped reduce stress-related damage and implement rapid recovery, as compared to IA plants (Couée et al., 2006; Keunen et al., 2013). Gibson (2005) also indicated that the sugar flux might act as a signal for the regulation of secondary metabolism. Indeed, soluble carbohydrates can stimulate the synthesis of phenylpropanoids (Bolouri-Moghaddam *et al*., 2010). Phenylpropanoids increased in both WS ecotypes but to a much higher extent in PY than IA leaves (Fig. 4C and D). We suggest that, especially in WS leaves of the PY ecotype, the soluble sugar increase upregulated the biosynthesis of phenylpropanoids that actively protect against reactive oxygen species (Tattini et al., 2015; Velikova *et al*., 2016; Bautista *et al.,* 2016).

Water stress had no effect on in Chl_Tot_ content in both ecotypes (Fig. 5). A similar response was previously observed for other species (Nikolaeva *et al*. 2010) as well as in young *M. oleifera* seedlings (Rivas *et al*., 2013) and likely reflects the fact that stress was too fast to cause pigment degradation (which was indeed observed only in the recovery phase, especially in PY plants). Maintenance of high levels of chlorophylls may have allowed faster recovery of photosynthesis after rehydration in both ecotypes and might have sustained synthesis of secondary pigments with photoprotective functions. While β-carotene was not affected in WS leaves (see above), water stress stimulated the biosynthesis of zeaxanthin and consequently enhanced the de-epoxidation status of xanthophylls pool (DES), particularly at midday, in IA leaves (Fig. 5). Thus, in the IA ecotype, water stress might have upregulated the plastid-localized 2-C-methyl-d-erythritol 4-phosphate (MEP) pathway, which also produces ABA. Accordingly, higher ABA and ABA-GE content were found in WS leaves of IA plants compared to PY plants (Fig. 6). In addition, a decrease in neoxanthin content was found at midday under stress, suggesting that ABA was produced from the cleavage of neoxanthin by the 9-cis-epoxycarotenoid dioxygenase (NCED), especially in IA leaves (Schwartz *et al*., 1997; Schwartz *et al*., 2003). While the different ABA formed in WS leaves of the two ecotypes seemingly did not influence *g*_s_ (equally reduced in PY and IA), it may be responsible for the reduced reopening of IA stomata once the stress was relieved (compare *g*_s_ of R-WS leaves of the two ecotypes in Fig. 1, when most of ABA was either scavenged or transformed in ABA-GE after re-watering). Higher ABA content may have accounted for the slightly but significantly higher RWC of IA leaves during the water stress treatment (Lu *et al.,* 2003; Flexas *et al.,* 2006).

After re-watering, many of the observed changes in the biochemistry of *M. oleifera* leaves during the WS treatment were reversed. However, in PY leaves sucrose and some secondary metabolites (e.g. kaempferol derivatives among phenylpropanoids and β-carotene) were produced at lower levels than in WW plants. Low sucrose may reveal a change in allocation of carbon to photosynthates after photosynthesis fully recovers from water stress. A higher synthesis of starch at the expense of sucrose could cause a feedback inhibition of photosynthesis (Sharkey, 2015) that would explain reduced Vc_max_ of IA and PY leaves at the R-WS stage. The capacity to quickly switch off the biosynthesis of secondary metabolites after recovery from stress may be a useful trait that allows PY ecotype to rapidly and fully restore photosynthesis and growth rates. After re-watering, IA ecotype is less able to restore the original rates of photosynthesis and growth after severe water stress, likely because of the high investment in quercetin derivatives with antioxidant function, to a (Fig. 4C).

### Phenotypic plasticity of the two ecotypes of *M. oleifera* from different climatic zones

Under water stress, plants of the PY ecotype showed a high degree of plasticity of some physiological (Vc_max_ and Φ_PSII_) and biochemical traits (content of total phenylpropanoids) (Table 1). On the other hand, IA plants plasticity was restricted to the MEP-derived isoprenoids, which was maintained after re-watering (see high NIV of ABA, ABA-GE, zeaxanthin and β-carotene in Table 1).

Such a pattern of plasticity has been reported for other plant species exposed to drought (Heschel *et al*., 2004; Ramírez-Valiente *et al*., 2010) and could reflect a different degree of tolerance to water stress of the two ecotypes of *M. oleifera* (Zhou *et al.*, 2015). In particular, both ecotypes showed a typical avoidance strategy (Levitt 1985; Franks *et al*., 2007); however, the PY ecotype, native to the humid subtropical region of Chaco Paraguayo, was characterized by a higher metabolic plasticity under drought and restored higher growth rates after re-watering compared to IA ecotype. On the other hand, IA ecotype, native to the tropical wet and dry region of Andhra Pradesh, although being able to cope with water stress, invested a high proportion of carbon into antioxidant metabolites under recovery, thus resulting in a typical trade-off between growth and defence.

In conclusion, *M. oleifera* demonstrated a high genetic variability and phenotypic plasticity, which will be key factors for adaptation to drought. The present study may be valuable to improve conservation, selection and collection of *M. oleifera* seeds from different regions, in order to develop breeding programs for the cultivation of this species in dry areas of the world. This would make *M. oleifera* an important resource for agro-forestry in a climate change scenario.

## Supplementary Material

Tab_SM_1_coaa028
