# Supplementary material for: Phenotypic plasticity of two M. oleifera ecotypes from different climatic zones under water stress and re-watering
Source: Conserv Physiol. 2020 Apr 13;8(1):coaa028. doi: 10.1093/conphys/coaa028 (PMC7154184; doi:10.1093/conphys/coaa028)
Supplement: Tab_SM_1_coaa028 [file tab_sm_1_coaa028.docx]

|  | π_tlp_ PY (MPa) | π_tlp_ IA (MPa) |
| --- | --- | --- |
| WW | -1,53 ± 0,022^a^ | -1,49 ± 0,103^a^ |
| WS | -1,66 ± 0,026^b^ | -1,64 ± 0,093^b^ |
| R-WW | -1,58 ± 0,052^a^ | -1,56 ± 0,013^a^ |
| R-WS | -1,48 ± 0,064^a^ | -1,55 ± 0,050^a^ |

Tab. SM1. Leaf turgor loss point (π_tlp_) in two *Moringa oleifera* ecotypes (PY ecotype from Paraguay and IA ecotype from India) comparing well-watered plants (WW) with plants at the end of water stress (WS), and well-watered plants (R-WW) with plants subjected to re-watering (R-WS). Data are means ± standard deviation (n=4). Different letters represent significant differences between ecotypes and treatments (*P*<0.05).
